# Characterization of Liaoning Cashmere Goat Transcriptome: Sequencing, *De Novo* Assembly, Functional Annotation and Comparative Analysis

**DOI:** 10.1371/journal.pone.0077062

**Published:** 2013-10-09

**Authors:** Hongliang Liu, Tingting Wang, Jinke Wang, Fusheng Quan, Yong Zhang

**Affiliations:** 1 College of Veterinary Medicine, Northwest A&F University, Key Laboratory of Animal Biotechnology of the Ministry of Agriculture, Yangling, Shaanxi, China; 2 School of Biological Science and Medical Engineering, Southeast University, Nanjing, Jiangsu, China; Wageningen UR Livestock Research, Netherlands

## Abstract

**Background:**

Liaoning cashmere goat is a famous goat breed for cashmere wool. In order to increase the transcriptome data and accelerate genetic improvement for this breed, we performed *de* *novo* transcriptome sequencing to generate the first expressed sequence tag dataset for the Liaoning cashmere goat, using next-generation sequencing technology.

**Results:**

Transcriptome sequencing of Liaoning cashmere goat on a Roche 454 platform yielded 804,601 high-quality reads. Clustering and assembly of these reads produced a non-redundant set of 117,854 unigenes, comprising 13,194 isotigs and 104,660 singletons. Based on similarity searches with known proteins, 17,356 unigenes were assigned to 6,700 GO categories, and the terms were summarized into three main GO categories and 59 sub-categories. 3,548 and 46,778 unigenes had significant similarity to existing sequences in the KEGG and COG databases, respectively. Comparative analysis revealed that 42,254 unigenes were aligned to 17,532 different sequences in NCBI non-redundant nucleotide databases. 97,236 (82.51%) unigenes were mapped to the 30 goat chromosomes. 35,551 (30.17%) unigenes were matched to 11,438 reported goat protein-coding genes. The remaining non-matched unigenes were further compared with cattle and human reference genes, 67 putative new goat genes were discovered. Additionally, 2,781 potential simple sequence repeats were initially identified from all unigenes.

**Conclusion:**

The transcriptome of Liaoning cashmere goat was deep sequenced, *de* *novo* assembled, and annotated, providing abundant data to better understand the Liaoning cashmere goat transcriptome. The potential simple sequence repeats provide a material basis for future genetic linkage and quantitative trait loci analyses.

## Introduction

Cashmere is an important commodity in China and Liaoning cashmere goat (*Capra hircus*) is famous for its fine cashmere quality, high cashmere yield, and stable genetic character. Although this population was bred in the 1980s, a little is known regarding the molecular mechanism and regulation of cashmere growth in this breed. Genetic studies of Liaoning cashmere goat had been performed in the past decade, which mainly focused on characterization and expression of functional genes [1,2] and development of genetic markers[3,4] for breeding and genetic evaluation. This is partly due to unavailable transcriptome information and the sequencing of limited number of randomly selected cDNA clones for Liaoning cashmere goat.

Expressed sequence tags (ESTs) or transcriptome sequencing is an efficient way to generate functional genomic-level data for an organism, especially non-model organism. ESTs provide comprehensive information about the transcriptome [5] and have played significant roles in investigating the level of gene expression [6,7], accelerating single nucleotide polymorphisms (SNPs) [8], microsatellites [9,10] and gene discovery [11-13], identifying alternative splicing[14], improving genome annotation, facilitating large-scale expression analysis, etc. However, constructing an EST database by traditional approaches was time-consuming and expensive. Recently, with the rapid development of the next-generation sequencing (NGS) technology, various platforms, such as Illumina Genome Analyzer, Roche 454 GS FLX and ABI SOLiD System, have provided a powerful and cost-effective tool for *de novo* transcriptome research. Especially, Roche 454 GS-FLX technology has generally been used for transcriptome analyses in a large number of animals [12-20], plants [10,21-24] and microorganisms [9,25-27].

In this article, we performed *de novo* transcriptome sequencing of the female Liaoning cashmere goat using Roche 454 GS FLX platform. 804,601 high-quality reads were assembled into a non-redundant set of 117,854 unigenes, comprising 13,194 isotigs and 104,660 singletons. Functional annotation and comparative analysis were performed on those 117,854 unigenes, providing an invaluable new resource for functional genomics and biological research in Liaoning cashmere goat. Finally, potential simple sequence repeats we identified could be useful for genetic linkage mapping and other genetic studies.

## Results and Discussion

### Transcriptome sequencing and assembly

Although the Roche 454 GS FLX platform incurs a high cost for sequencing, the read length of the output sequences are more than adequate for the *de novo* assembly of Liaoning cashmere goat genes [28,29]. To achieve a whole-body transcriptome, total RNA was extracted from a variety of adult organs and tissues including heart, liver, spleen, lung, kidney, pancreas, skin, muscle, ovary, stomach, intestines, brain and arteries. Equal quantities of total RNA were mixed together to construct a cDNA library. The library was sequenced by a Roche 454 GS FLX.

**Figure 1:**
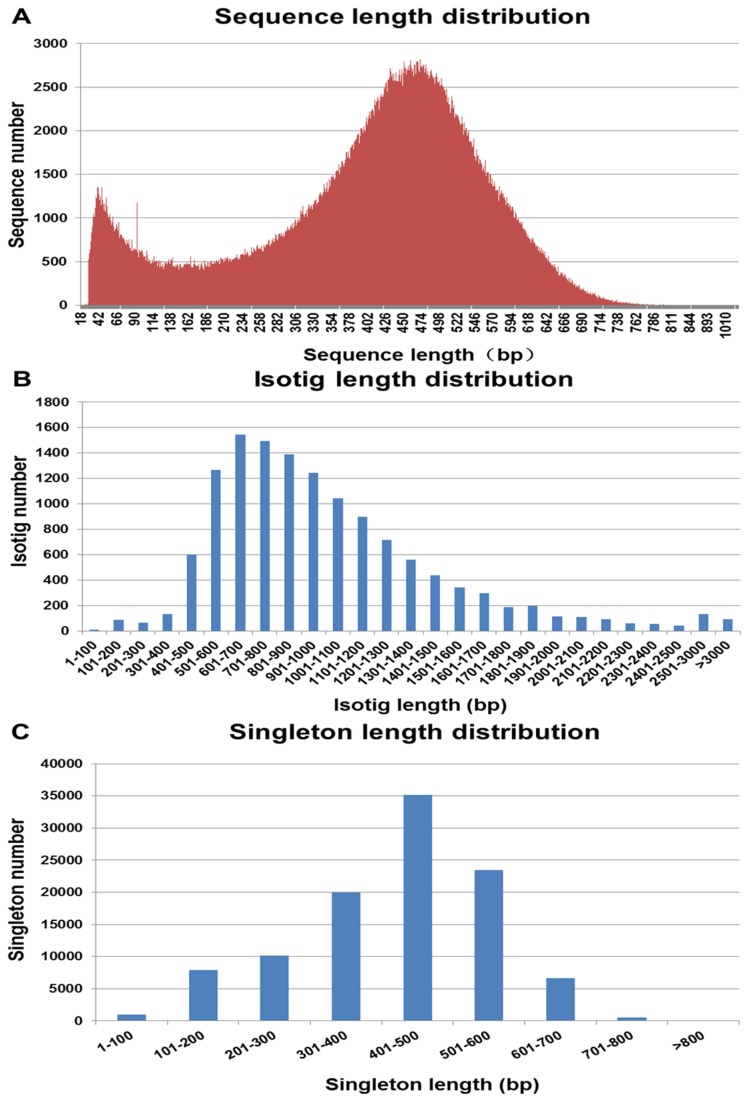
Length distribution of Liaoning cashmere goat transcriptomic sequences. (**A**) total transcriptomic reads, (**B**) isotigs, (**C**) singletons.

Table 1 shows the summary of Roche 454 GS FLX assembly and analysis of transcriptomic sequences in Liaoning cashmere goats. A total of 1,044,032 raw reads were obtained with an average length of 416 bps. After processing the raw reads and filtering out short and low quality reads, 804,601 (77.07%) high-quality transcriptomic reads with an average size of 403 bp remained (Figure 1A). De novo assembly using Newbler Software v. 2.5 generated 13,194 isotigs (comprising 654,169 reads (81.30%)). The isotig length ranged from 91 to 7,402 bp (Figure 1B), with an average length of 1,011 bp. Most isotigs (93.24%) were > 500 bp in length, and 40.64% of them were > 1000 bp. The average length of the Liaoning cashmere goat isotigs (1,011 bp) was longer than most of those assembled in other non-model organisms, such as *Melitaea cinxia* (197 bp) [30], *Acropora millepora* (440 bp) [31], *P. contorta* (500 bp) [32], *Pristina leidyi* (707 bp) [33], and *Megalobrama amblycephala* (730 bp) [19], but they were shorter than the average length in *Junco hyemalis* (1,248 bp) [34]. About 112,148 (18.70%) reads did not assembled into isotigs. After processing these, 104,660 singletons with an average length of 422 bp were obtained. The singletons ranged from 90-1,205 bp, and 98.62% of the singletons were between 101 and 700 bp in length (Figure 1C). Finally, clustering and assembly of 804,601 high-quality reads produced a non-redundant set of 117,854 unigenes, comprising 13,194 isotigs and 104,660 singletons. 

**Table 1:** Summary of Roche 454 GS FLX assembly and analysis of Liaoning cashmere goat transcriptomic sequences.

**Data generation and filtering**	
Number of total reads	1,044,032
Average read length (bp)	416
Range read length (bp)	18-1,570
Number of high quality reads (after filtering)	804,601(77.07%)
Average read length (bp) (after filtering)	403
Range read length (bp) (after filtering)	18-1,483
**Assembly statistics**	
Number of reads assembled	654,169 (81.30%)
Number of isotigs	13,194
Average isotig length (bp)	1,011
Range isotig length (bp)	91-7,402
Number of isotig length>500 bp	12,302(93.24%)
Number of isotig length>1000 bp	5,362 (40.64%)
Number of reads unassembled	112, 148(18.70%)
Number of singletons	104,660
Average singleton length (bp)	422
Range singleton length (bp)	90-1,205

### Functional annotation and classification

All unigenes (isotigs and singletons) were analyzed by EMBOSS software [35] for generation of putative protein sequences. Then we searched all the putative protein sequences against several protein databases (NCBI non-redundant (NR), Swiss-Prot and TrEMBL, Clusters of Orthologous Groups (COG), and Kyoto Encyclopedia of Genes and Genomes (KEGG) ), and predicted the protein functions from the annotations of the most similar proteins. Here, 53,515 (45.41%) unigenes were annotated (Table 2).

**Table 2:** Summary statistcs of functional annotation for Liaoning cashmere goat unigenes in public protein databases.

**Public protein database**	**Number of unigene hits**	**Percentage (%)**
**NR**	24,614	20.89
**Swiss-Prot and TrEMBL**	17,356	14.73
**COG**	46,778	39.69
**KEGG**	3,548	3.01
**Total**	53,515	45.41

All putative protein sequences were subjected to a BLASTp similarity search against the non-redundant protein database of NCBI (www.ncbi.nlm.nih.gov), with a cutoff E value of <1e^- 5^. 24,614 unigenes had significant BLASTp matches, including 5,340 isotigs (40.47%) and 19,274 singletons (18.42%). Similarly low matching percentages of isotigs and singletons have been reported in non-model species, such as in *Macrobrachium nipponense* (40.76% contigs and 17.71% singletons) [19], *Junco hyemalis* (49% isotigs and 11% singletons) [34], and *Scophthalmus maximus* (44.84% contigs and 7.55% singletons) [20]. 

**Figure 2:**
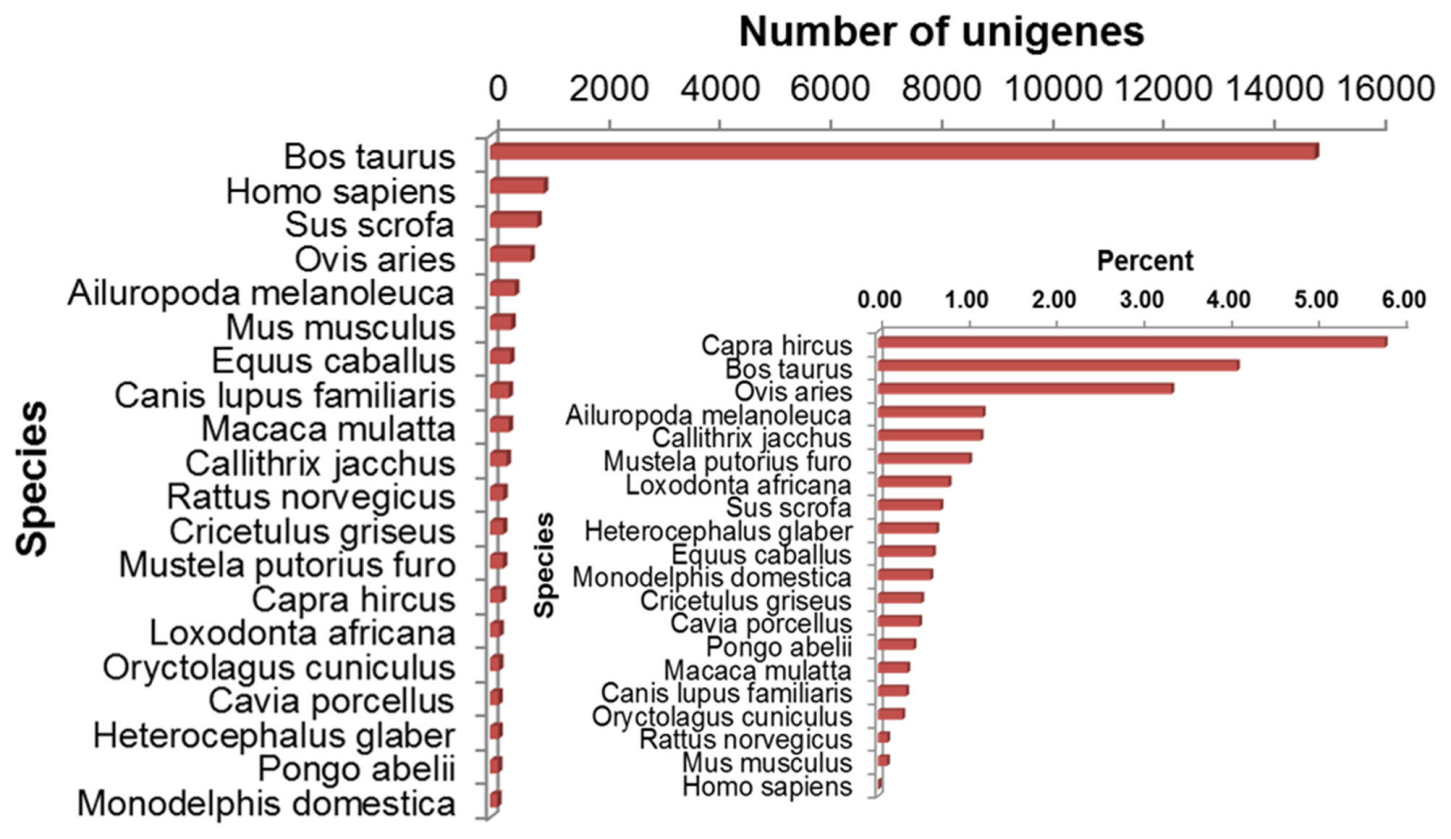
Statistical analysis of matched sequences from the Liaoning cashmere goat. The clear bars represent the top-hit species distribution of BLAST matches of Liaoning cashmere goat unigenes. Note that nearly 68% of top hits are to *Bos* *taurus*, whose complete genome has been sequenced. The shaded bars (inset) represented the percent of matched unigenes in the total sequences of the NCBI Nr database of each species in the top 20. Nearly 6% of the *Capra* *hircus* sequences in NCBI NR database are covered.

Among the annotated 24,614 unigenes, the majority of matched sequences exhibited high similarity to *Bos taurus* (60.37%) sequences. The top 20 species distribution of BLAST matches is shown in Figure 2. The annotated unigenes of the Liaoning cashmere goat transcriptome have a low percentage of matches to *Capra hircus* (goat) or *Ovis aries* (sheep) sequences. In principle, the unigenes of the Liaoning cashmere goat should be more similar to these two species, because they all belong to the *Caprinae*. We speculated that this might have resulted from an underrepresentation of goat and sheep protein sequences in the NCBI Nr database. To confirm this speculation, we collected the total number of protein sequences in NCBI Nr database for the top 20 species, then calculated the percent of matched unigenes in the total sequences of NCBI Nr database for each species in the top 20 (Figure 2, inset). As a result, it was found that the matched unigenes of the Liaoning cashmere goat exhibited the highest similarity to *Capra hircus* sequences. This validated our speculation regarding the underrepresentation of goat and sheep protein sequences. 

**Figure 3:**
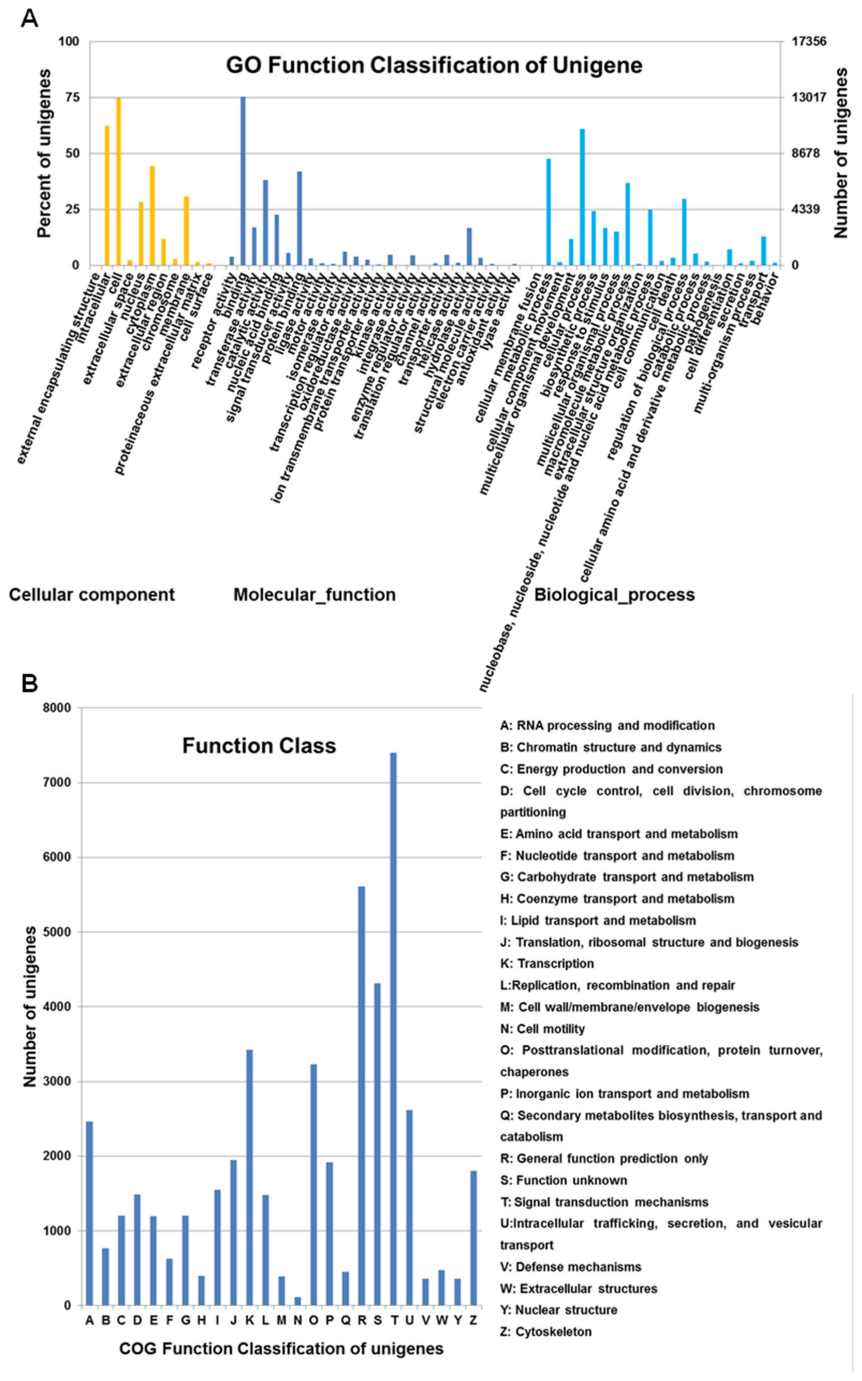
GO and COG classifications of unigenes derived via Roche 454 sequencing of the Liaoning cashmere goat. (**A**), GO classification of the Liaoning cashmere goat unigenes. 17,356 unigenes were assigned to 6,700 GO categories, and the terms were summarized into the three main GO categories and 59 sub-categories. Right y-axis, percentage of unigenes; left y-axis, number of unigenes. (**B**), COG Function Classification of the Liaoning cashmere goat unigenes. 46,778 unigenes showing significant homology to COG database at NCBI had COG classification among 25 categories.

Gene ontology (GO) is an international classification system for standardized gene functions, offering a comprehensive description of the gene properties and their products in any organism. All putative protein sequences (obtained from 117,854 unigenes) of Liaoning cashmere goat were assigned for GO terms based on sequence similarity, using BLASTp (with E value<1e^-5^) against the Swiss-Prot and TrEMBL database, and then using the GoPipe software[36] to perform GO functional classifications. In total of 17,356 unigenes were assigned to 6,700 GO categories, and the terms were summarized as three main GO categories and 59 sub-categories (Figure 3A). Of these, 15,288 (88.08%) comprised the largest category, molecular function, followed by cellular component (14,617, 84.22%) and biological process (14,119, 81.35%). Among the molecular functions category, the top three were involved in binding (GO: 0005488), protein binding (GO: 0005515) and catalytic activity (GO: 0003824). Regarding cellular components, cell (GO: 0005623) and intracellular (GO: 0005622) were the dominant groups, cytoplasm (GO: 0005737) and membrane (GO: 0016020). Within biological processes category, cellular process (GO: 0009987) was the most dominant group, metabolic process (GO: 0008152), macromolecule metabolic process (GO: 0043170) and regulation of biological process (GO: 0050789) (Table **S1**). 

Cluster of Orthologous Groups (COG) is a database where orthologous gene products may be classified. All unigenes were subjected to a search against the COG database for functional prediction and classification. Some unigenes had multiple COG functions. 46,778 unigenes showing significant homology to entries in the COG database at NCBI were classified into 25 COG categories (Figure 3B). Among the 25 COG categories, the largest group was “signal transduction mechanisms” with 15.82% unigenes, followed by “general function prediction only” (11.99%), “function unknown” (8.11%), “transcription” (7.31%), “posttranslational modification, protein turnover, chaperones” (6.90%), and “intracellular trafficking, secretion, and vesicular transport” (5.60%). The smallest groups were “cell motility” (0.24%) and “defense mechanisms” (0.77%).

To better understand the biological functions and interactions of the putative proteins (obtained from 117,854 unigenes), a bidirectional BLAST search against Kyoto Encyclopedia of Genes and Genomes (KEGG) protein database with a cutoff E value of <1e^- 5^ was performed. 3,548 unigenes, comprising 1,728 isotigs and 1,820 singletons, had KEGG Orthology (KO) numbers (Table **S2**). According to the KO numbers, we assigned the 3,548 unigenes to 300 KEGG pathways (Table **S2**). The pathways represented by the KEGG annotated unigenes were in cancer (ko05200), HTLV-I infection (ko05166), RNA transport (ko03013), Epstein-Barr virus infection (ko5169), protein processing in endoplasmic reticulum (ko04141) and MAPK signaling pathway (ko04010).

In addition, the 3,548 KEGG annotated unigenes could be categorized into six different functional groups (Table 3). The largest group is metabolism, which contained 1,117 KEGG annotated unigenes. This KEGG category consisted of 11 major sub-groups, such as carbohydrate metabolism, amino acid metabolism, lipid metabolism. Other KEGG categories were human diseases, genetic information processing, organismal systems, cellular processes and environmental information processing. A well-categorized and annotated transcriptome would be useful for further investigations of gene function in future studies.

**Table 3:** KEGG classification of Liaoning cashmere goat unigenes.

**KEGG categories**	**No. of unigenes**	**KEGG categories**	**No. of unigenes**
**Metabolism**	**1,117**	**Environmental Information Processing**	**679**
Carbohydrate Metabolism	246	Membrane Transport	33
Energy Metabolism	185	Signal Transduction	459
Lipid Metabolism	212	Signaling Molecules and Interaction	251
Nucleotide Metabolism	136	**Organismal Systems**	**982**
Amino Acid Metabolism	216	Immune System	423
Metabolism of Other Amino Acids	79	Endocrine System	236
Glycan Biosynthesis and Metabolism	145	Circulatory System	88
Metabolism of Cofactors and Vitamins	143	Digestive System	168
Metabolism of Terpenoids and Polyketides	29	Excretory System	78
Biosynthesis of Other Secondary Metabolites	18	Nervous System	220
Xenobiotics Biodegradation and Metabolism	85	Sensory System	30
**Genetic Information Processing**	**985**	Development	121
Transcription	166	Environmental Adaptation	21
Translation	358	**Human Diseases**	**1,034**
Folding, Sorting and Degradation	376	Cancers	247
Replication and Repair	127	Immune Diseases	104
**Cellular Processes**	**739**	Neurodegenerative Diseases	213
Transport and Catabolism	329	Substance Dependence	77
Cell Motility	99	Cardiovascular Diseases	104
Cell Growth and Death	205	Endocrine and Metabolic Diseases	38
Cell Communication	220	Infectious Diseases	613
		**Total**	**3,548**

### Comparative analysis

#### Gene Coverage Analysis

**Figure 4:**
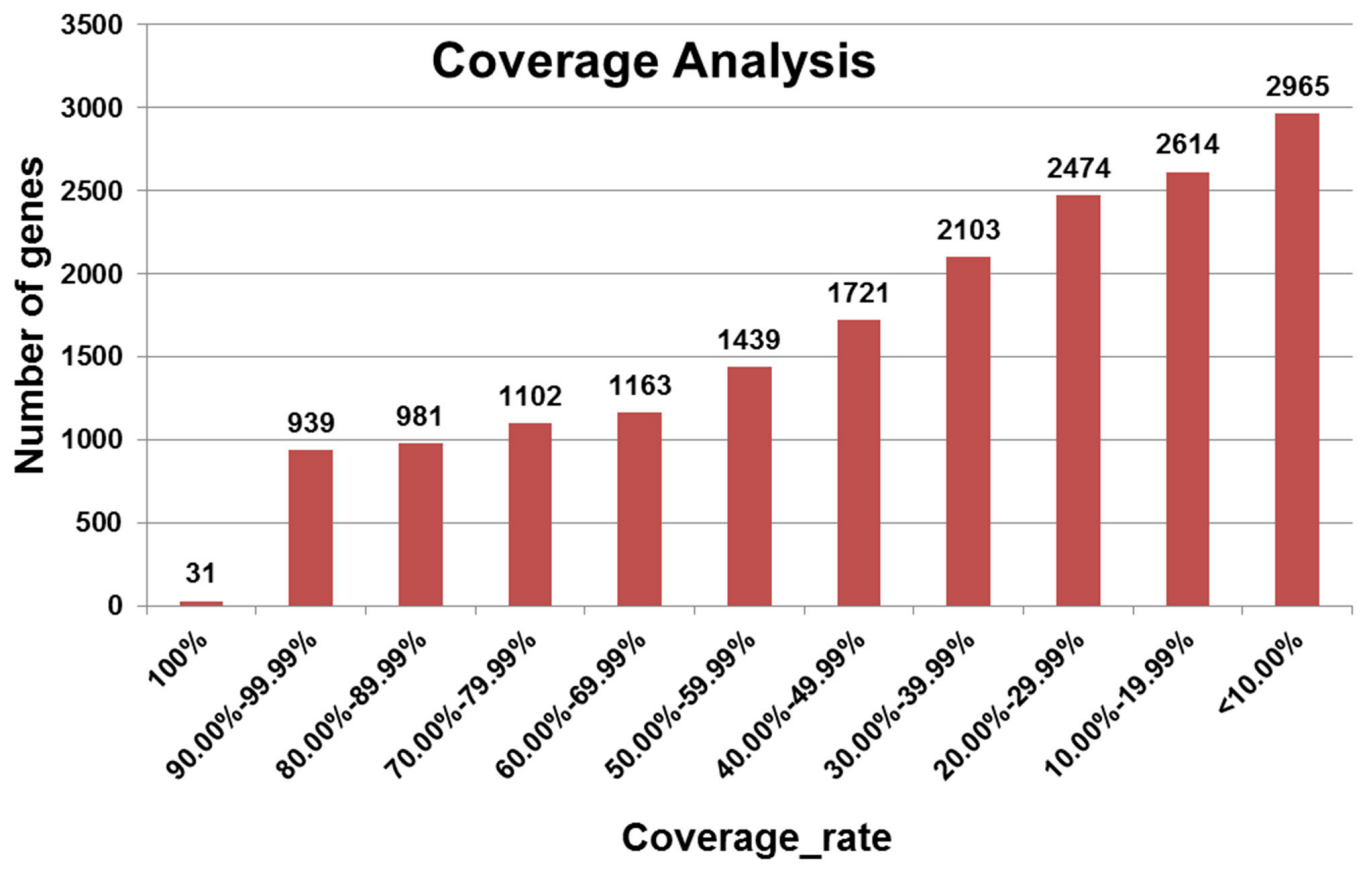
Coverage analysis of genes identified by BLAST searching against NCBI non-redundant nucleotide databases. 42,254 unigenes align to 17,532 different specific genes.

After clustering and assembling 804,601 high-quality reads, we obtained 117,854 non-redundant unigenes, comprising 13,194 isotigs and 104,660 singletons. The assembled unigenes were compared with the sequences in the NCBI non-redundant nucleotide databases using the BLASTn algorithm with a cutoff E value of <1e^-20^. Of the 117,854 assembled unigenes, 66,351 (56.30%) showed one alignment to an existing sequence. After processing and filtering, 42,254 unigenes were aligned to 17,532 different specific genes. The coverage of every gene was also calculated (Table **S3**). Among the 17,532 specific genes, 31 genes had full-length coverage, and 939 (5.36%) genes had coverage between 90.00% and 99.99%. Moreover, 3,053 (17.41%) genes had a coverage > 70%, and 5,655 (32.26%) genes had a coverage > 50% (Figure 4). 

According to the result of the gene coverage analysis, the full-length sequences of some Liaoning cashmere goat genes could be obtained by using PCR or reverse PCR. The 42,254 unigenes aligned to specific genes could serve as a library, facilitating the isolation of the full-length versions of Liaoning cashmere goat genes. Using the genes that participate in hair follicle formation and hair growth regulation as example, we identified *FGF7*, *KAP8.1*, *Hoxc13*, *TGFβ*, *PDGFA*, *IGFBP5* and *BMP4* as having a coverage rate >50% in Table **S3**. In addition, our data contain a number of other genes related to hair follicle formation and hair growth regulation with a coverage rate <50%, such as *TGFβ2*, *FGF5*, *Notch1*, *IGF-1*, *PDGFRA*, *BDNF*, *IL1A/B*, *NTRK3*, and *MTNR1A*. Without doubt, there were also many genes that were not represented in this RNA-seq dataset, because of the depth of 454 sequencing, the sources of the sequencing samples and low transcript abundance.

To date, although 2.66-Gb genome sequences of a female Yunnan black goat have been reported[37], the comprehensive annotation of goat genome remains unavailable. Thus, we believe that our sequence library (comprised 42,254 unigenes) would serve as an important public information platform to accelerate functional gene studies in Liaoning cashmere goat. For example, the sequence library could facilitate studies of genes, gene transcription and transcriptome of other goat species by providing an abundant source of homologous genes.

#### Comparison with *Capra hircus* genome sequence and protein-coding genes

*Capra hircus* genomes information is publicly available through a genome browser interface and database (http://goat.kiz.ac.cn/GGD/). To orient the Liaoning cashmere goat unigenes derived from RNA-seq, we performed BLASTn alignment (E value <1e^-20^) against the sequences of *Capra hircus* genomes. Among a total 117,854 unigenes, 97,236 (82.51%) were mapped to 30 chromosomes of *Capra hircus* (Table **S4**). Furthermore, all matched unigenes (97,236) were compared with the 66,351 annotated unigenes, which showed at least one significant alignment to an existing sequence in the NCBI non-redundant nucleotide databases. 53,140 (54.65%) matched unigenes got annotated. This analysis indicated that our assembled unigenes obtained from 13 Liaoning cashmere goat tissues will be useful for the annotation of *Capra hircus* chromosomes.

In addition, all Liaoning cashmere goat unigenes were searched against 22,175 *Capra hircus* protein-coding genes, which have been reported[37] and were publicly available (http://goat.kiz.ac.cn/GGD/). 35,551 (30.17%) unigenes, comprising 6,935 isotigs and 28,616 singletons, were mapped to 11,438 (51.58%) *Capra hircus* protein-coding genes (Table **S5**). To further annotate and identify unigenes which were not matched to the 22,175 *Capra hircus* protein-coding genes, we mapped all non-matched unigenes to *Bos taurus* (cattle) and *Homo sapiens* (human) reference genes, using the BLASTn algorithm with a cutoff E value of <1e^-20^. Of 82,303 non-matched unigenes, 15,779 were aligned to 6,060 cattle reference genes, and 6,837 were aligned to 2,880 human reference genes (Table **S6**). By merging the results, 18,602 (15.78% of all unigenes) unigenes were matched to 8,940 cattle and human reference sequences, as multiple unigenes may be aligned to one reference sequence. Together with the 35,551 unigenes which were mapped to *Capra hircus* protein-coding genes, amounting to 54,153 (45.95%) unigenes were annotated, including 9,289 (70.40%) isotigs and 44,864 (42.87%) singletons. This result will greatly increase the number of annotated sequences for the Liaoning cashmere goat in the NCBI database. 

#### Discovery of putative novel goat genes

The homology-based annotation is a serviceable method for gene prediction and annotation. Thus, we further analyzed the result of above comparative analysis, especially the 18,602 unigenes which were not mapped to the reported *Capra hircus* protein-coding genes but matched to 8,940 cattle and human reference genes. After statistical investigation, among the 8,940 cattle and human reference genes, 272 genes whose full-length were covered more than 50% by our Liaoning cashmere goat unigenes. Moreover, we collected coding sequences (CDS) of the 272 genes, and through comparative analysis, 67 cattle genes whose coding sequence were completely covered by 67 unigenes (Table **S7**). We predicted that the 67 Liaoning cashmere goat unigenes were homologous genes of the 67 cattle genes. Thus, we infer that the 67 Liaoning cashmere goat unigenes, which were not aligned to the 22,175 reported goat genes, were putative novel goat genes (Table **S8**). 

### Putative SSR markers in unigenes

Simple sequence repeats (SSRs) are polymorphic loci present in genomic DNA, and are distributed in both coding and non-coding regions [38]. They consist of repeated core sequences of 2-6 base pairs in length. We used MIcroSAtellite (MISA, http://pgrc.ipk-gatersleben.de/misa/) to identify potential SSRs in our unigenes. Of the 117,854 unigenes obtained from 454 sequencing, 2,621 unigenes were identified to contain 2,781 SSRs, 122 of the unigenes contained two or more SSRs. The frequency of SSR occurrence in the 117,854 unigenes was 2.36%. Table 4 shows that the dinucleotide repeats were the most common SSRs in our dataset. The second major class was pentanucleotide, and the remaining repeat motifs were mononucleotide, trinucleotide, tetranucleotide and hexanucleotide. This distribution was different from SSRs of *Capra hircus* skin[39], whose trinucleotide repeats were the most common type (38.6%) and the proportion of pentanucleotide repeats were small. In addition, the frequencies of SSRs with different number of tandem repeats are also shown in Table 4. SSRs with > 10 tandem repeats (49.08%) were the most common, followed by four tandem repeats, five tandem repeats and ten tandem repeats. The dominant repeat motif in all SSRs was AC/GT (908, 37.49%), followed by AACTG/AGTTC (326, 13.46%), AT/AT (211, 8.71%), AGC/CTG (137, 5.66%) and AAAT/ATTT (92, 3.80%) (Table **S9**). The AC/GT repeat type, most abundant in Liaoning cashmere goat was also very abundant in other vertebrates[40], but different from plants[41]. 

**Table 4:** Summary of SSRs from Liaoning cashmere goat unigenes.

**Motif length**	**Repeat numbers**								**Total**	**Percent**
	**4**	**5**	**6**	**7**	**8**	**9**	**10**	**>10**		
Mono-	-	-	-	-	-	-	-	359	359	12.91
Di-	-	-	-	-	-	-	233	963	1196	43.01
Tri-	-	-	-	150	62	42	32	32	318	11.43
Tetra-	-	200	65	16	3	4	1	9	298	10.72
Penta-	454	78	12	2		1		0	547	19.67
Hexa-	48	5	8					2	63	2.27
**Total**	502	283	85	168	65	47	266	1365		
**Percent**	18.05	10.18	3.06	6.04	2.34	1.69	9.56	49.08		

SSRs can be used for subsequent marker development, genetic linkage and quantitative trait loci analysis. To make these SSR markers be useful for Liaoning cashmere goat breeding or other studies, we employed primer 3 to design primer pairs for each SSR. After removing the microsatellites without sufficient flanking sequence for primer design, 958 primer pairs from 2,781 (34.45%) SSRs were designed (Table **S10**). However, these primers require further experimental validation.

## Conclusions

In this study, *de novo* transcriptome sequencing of the female Liaoning cashmere goat using the 454 GS FLX system was performed for the first time. 1,044,032 raw reads were *de novo* assembled into 117,854 unigenes. All unigenes were then evaluated and functionally annotated by comparing with the existing protein databases, such as NCBI NR database, Swiss-Prot and TrEMBL database, COG database, and KEGG protein database. Further comparative analysis revealed that 54,153 (45.95%) unigenes got annotated and 67 putative new goat genes were discovered. Moreover, 2,781 SSRs were initially identified among the unigenes, which can be used for subsequent marker development, genetic linkage and quantitative trait loci analysis. Overall, our study on the Liaoning cashmere goat transcriptome provides a valuable resource that will lead to a better understanding of the Liaoning cashmere goat transcriptome.

## Materials and Methods

### Ethics statement

All animal procedures and study design were conducted in accordance with the Guide for the Care and Use of Laboratory Animals (Ministry of Science and Technology of China, 2006), and were approved by the animal ethics committee of Northwest A & F University.

### Sample preparation, total RNA isolation and quality controls

The most important issue for transcriptome sequencing was the preparation of fresh and healthy tissue samples. Therefore, three adult female Liaoning cashmere goats were selected from Liaoning Province of China in 2012. The 13 tissue samples including heart, liver, spleen, lung, kidney, pancreas, skin, muscle, ovary, stomach, intestines, brain and arteries were freshly collected. All samples were immediately frozen in liquid nitrogen and stored at -80°C until use.

Total RNA was extracted from the 13 tissues using the TRIzol Reagent (Invitrogen, USA) according to the manufacturer’s instructions. DNase I (Ambion, USA) was used to remove potential genomic DNA contamination from the RNA samples. The quality of the total RNA was determined using a Nanodrop spectrophotometer (Thermo, USA). Only RNA samples with an OD_260_:OD_280_ ratio of 1.9- 2.1 and an OD_260_:OD_230_ ratio of 2.0 -2.5 were used for subsequent analysis. Integrity and size distribution were checked with an Agilent 2100 Bioanalyzer (Agilent, USA) RNA 6000 Pico LabChip (RIN value). 

### cDNA synthesis and 454 sequencing

We pooled equivalent amounts of total RNA from each sample (total 0.30 mg total RNA) and delivered it to Shanghai Hanyu Bio-Tech Co., Ltd. (Shanghai, China) for cDNA synthesis and 454 sequencing. Pure mRNA was subtracted using the Micropoly(A) Purist^TM^ mRNA purification kit (Ambion). RNA quality and quantity was determined again at the end of this process. First-strand cDNA was synthesized using GsuI-oligo dT as reverse transcription primers, 10 mg of the mRNA as template, and 1000 units of Superscript II reverse transcriptase (Invitrogen), at 42°C for 1 h. The mRNA 5' cap structure was then oxidized using NaIO_4_ (Sigma, USA) and connected to biotin. The biotin-coupled mRNA/cDNA was screened by Dynal M280 beads (Invitrogen), and the first-strand cDNA was released by alkaline dissociation. Subsequently, adapters were added to the first-strand cDNA using DNA ligase (TaKaRa, Japan), and the second-strand cDNA was synthesized using Ex Taq polymerase (TaKaRa). Finally, the polyA and 5' end adapters were removed by Gsu I digestion.

Fragmentation of synthesized cDNA was performed by nebulization, which shears double-stranded cDNA into fragments ranging from approximately 300 to 800 base pairs. A single-strand template DNA (sstDNA) library was constructed using a GS DNA Library Preparation kit (Roche Applied Science, USA) following the manufacturer’s protocol. The sstDNA was attached to beads using a GS emPCR kit (Roche Applied Science), and an emulsion-based clonal amplification was performed. After amplification, Roche GS-FLX 454 pyrosequencing was conducted, according to the manufacturer’s recommendations.

### Sequence assembly

1,044,032 raw sequences were obtained from Roche 454 pyrosequencing, with an average length of 416 bp. Newbler Software v. 2.5 (provided with the Roche GS FLX sequencer) was used for sequence assembly, using default parameters. Prior to assembly, we removed the nonsense sequences, including adapters added for reverse transcription and 454 sequencing, primers and low quality sequences, using Newbler. Raw data were deposited in NCBI Sequence Read Archive (SRA) under the accession number SRA064946. All unigene sequences (>200 bp) were deposited to the Transcriptome Shotgun Assembly (TSA) database. This Transcriptome Shotgun Assembly project has been deposited at DDBJ/EMBL/GenBank under the accession GAFC00000000. The version described in this paper is the first version, GAFC01000000.

### Functional annotation

All unigenes were analyzed by the EMBOSS software package [35] for generation of putative protein sequences. All putative protein sequences were subjected to a BLASTp similarity search against the NR protein database (NCBI), with a cutoff E value of <1e^-5^. Blast hits annotated with uninformative terms such as "unknown", "uncharacterized" and "hypothetical" were omitted from analyses. Ultimately, 24,614 unigenes had significant BLASTp matches. 

To annotate unigenes with gene ontology (GO) terms, sequences were compared against the Swiss-Prot and TrEMBL database using the BLASTp algorithm, with a cutoff E value of <1e^-5^. The results were then sorted by GO categories using gene2go in the GoPipe software [36]. BLASTp was also used to match unigenes to the Clusters of Orthologous Groups (COG) (http://www.ncbi.nlm.nih.gov/COG/) to predict possible functional classifications. KEGG Orthology (KO) numbers were assigned to unigenes using a bidirectional BLAST search against the KEGG protein database with a cutoff E value of <1e^-5^. The sequences were mapped to the KEGG pathways according to the KO numbers in the pathway database. 

### Calculation of gene coverage

The assembled sequences were compared with the sequences in the NCBI non-redundant nucleotide databases using the BLASTn algorithm with a cutoff E value of <1e^-20^. 42,254 unigenes aligned to 17,532 different specific genes, because multiple unigenes sometimes matched to one specific gene. We collected the full-length versions of the 17,532 different specific genes, and then calculated the length of every specific gene covered by the 42,254 unigenes. The results of the coverage analysis are shown in Table **S3**.

### Comparative analysis

To further annotate and identify the assembled sequences, all the sequences (13,194 isotigs and 104,660 singletons) were mapped to *Capra hircus* genome and compared with the reported 22,175 *Capra hircus* protein-coding genes, using BLASTn with a cutoff E value of <1e^-20^. The remaining non-matched unigenes were also aligned to *Bos taurus* (cattle) and *Homo sapiens* (human) reference genes, using BLASTn with a cutoff E value of <1e^-20^. 18,602 unigenes were matched to 8,940 cattle and human reference genes. Among the 8,940 cattle and human reference genes, 272 genes whose full-length were covered more than 50% by unigenes. To discover the putative new goat genes, the coding sequences (CDS) of 272 cattle and human reference genes collected from NCBI. Through analysis, 67 genes whose coding sequence were completely covered by 67 Liaoning cashmere goat unigenes.

### SSR detection and primer designing

SSRs were detected among the 117,854 unigenes using MIcroSAtellite (MISA, http://pgrc.ipk-gatersleben.de/misa/), which accepts FASTA-formatted sequence files. The parameters were adjusted to identify perfect mono-, di-, tri-, tetra-, penta-, and hexanucleotide motifs with a minimum of 20, 10, 7, 5, 4 and 4 repeats, respectively. The maximum number of bases interrupting two SSRs in a compound microsatellite was set at 100. Primer 3 software [42] was used to design primers. The file “FASTAfile.misa”, which was created by MISA, contained the information about the type and the localization of each individual microsatellite and was submitted to Primer3 for primer designing. The major parameters for designing the primers were set as follows: primer length ranging from 18 to 22 bases, with 20 as the optimum; PCR product size ranging from 100 to 500 bp; optimum annealing temperature 59°C; and GC content from 40% to 70%, with 50% as the optimum.

## Supporting Information

Table S1 **Categories of gene ontology of Liaoning cashmere goat unigenes.**
(XLSX)

Table S2 **KEGG summary of Liaoning cashmere goat unigenes.**
(XLSX)

Table S3 **The statistics of gene coverage rate.**
(XLSX)

Table S4 **The result of Liaoning cashmere goat unigenes mapped to goat genome sequence.**
(XLSX)

Table S5 **The result of Liaoning cashmere goat unigenes aligned to 22,175 goat protein-coding genes.**
(XLSX)

Table S6 **The result of non-matched unigenes compared with cattle and human reference genes.**
(XLSX)

Table S7 **The analysis result of 67 Liaoning cashmere goat unigenes compared with 67 cattle reference genes.**
(XLSX)

Table S8 **Summary of the 67 putative novel goat genes.**
(DOCX)

Table S9 **Frequency statistics of classified repeat types (considering sequence complementary).**
(XLSX)

Table S10 **Summary of 958 SSRs and their primers.**
(XLSX)
