# Supplementary material for: Characterization of Liaoning Cashmere Goat Transcriptome: Sequencing, De Novo Assembly, Functional Annotation and Comparative Analysis
Source: PLoS One. 2013 Oct 9;8(10):e77062. doi: 10.1371/journal.pone.0077062 (PMC3793953; doi:10.1371/journal.pone.0077062)
Supplement: Table S8 — Summary of the 67 putative novel goat genes. (DOCX) [file pone.0077062.s008.docx]

**Table S8 Summary of the 67 putative novel goat genes**

| Unigenes | Accession number | E-value | Description |
| --- | --- | --- | --- |
| isotig14752 | XM_869219.3 | 0 | oligosaccharyltransferase 4 homolog |
| isotig14705 | NM_001113317.1 | 0 | split hand/foot malformation (ectrodactyly) type 1 |
| isotig14566 | NM_174623.2 | 0 | thymosin beta 10 |
| isotig14420 | XM_001789566.2 | 1.00E-137 | uncharacterized LOC784537 |
| isotig14338 | NM_176855.1 | 0 | oxytocin, prepropeptide |
| isotig13968 | NM_001033608.1 | 0 | macrophage migration inhibitory factor |
| isotig13756 | NM_174811.3 | 0 | guanine nucleotide binding protein (G protein), gamma 5 |
| isotig13704 | XM_001251646.3 | 0 | uncharacterized LOC786364 |
| isotig13558 | NM_001190300.1 | 1.00E-150 | microseminoprotein, beta |
| isotig13555 | XM_001790361.1 | 0 | uncharacterized LOC100140375 |
| isotig13542 | XM_002703847.1 | 0 | uncharacterized LOC100335236, transcript variant 1 |
| isotig13456 | NM_001101225.1 | 0 | polymerase (DNA-directed), epsilon 4 |
| isotig13158 | NM_174707.2 | 0 | matrix Gla protein |
| isotig13056 | XM_002702202.1 | 7.00E-96 | uncharacterized LOC100297004 |
| isotig13031 | NM_001034787.1 | 0 | mitochondrial ribosomal protein 63 |
| isotig12994 | NM_001075542.1 | 0 | cornifelin |
| isotig12157 | XM_002696332.1 | 0 | cytochrome c oxidase subunit 8A, mitochondrial-like, transcript variant 1 |
| isotig12126 | NM_001099204.1 | 0 | mitotic spindle organizing protein 2B |
| isotig11944 | NM_001077520.1 | 0 | cysteine-rich C-terminal 1 |
| isotig11929 | NM_001206365.1 | 0 | sarcolipin |
| isotig11335 | NM_001113230.1 | 0 | acylphosphatase 2, muscle type |
| isotig11250 | NM_001163139.1 | 0 | glutathione peroxidase 2 |
| isotig10848 | NM_001205995.1 | 0 | WW domain binding protein 5 |
| isotig10641 | NM_174320.3 | 1.00E-180 | FXYD domain containing ion transport regulator 2 |
| isotig10383 | NM_001077893.1 | 0 | eukaryotic translation initiation factor 4E binding protein 1 |
| isotig09998 | NM_001015531.1 | 0 | ribosomal protein S5 |
| isotig09479 | NM_174076.3 | 0 | glutathione peroxidase 1 |
| isotig09186 | NM_001144087.1 | 0 | coiled-coil domain containing 85B |
| isotig09136 | NM_001034352.1 | 0 | bolA homolog 1 |
| isotig08802 | NM_001110446.1 | 0 | DiGeorge syndrome critical region gene 6 |
| isotig08395 | NM_001007815.1 | 0 | microsomal glutathione S-transferase 1 |
| isotig07893 | NM_001035500.1 | 0 | mitochondrial ribosomal protein S34 |
| isotig07577 | NM_001046157.1 | 0 | spondin 2 |
| isotig07051 | NM_001045996.1 | 0 | mitochondrial ribosomal protein L28 |
| isotig05514 | NM_001113313.1 | 0 | neurogranin |
| isotig04462 | NM_001077903.1 | 0 | cyclin-dependent kinase inhibitor 1C |
| isotig04217 | NM_001015536.1 | 0 | thioredoxin domain containing 12 |
| isotig03636 | NM_001015567.2 | 0 | serine/threonine kinase receptor associated protein |
| isotig03456 | NM_001013583.1 | 0 | carboxyl ester lipase |
| isotig03408 | XM_001254067.2 | 0 | uncharacterized LOC786372 |
| isotig03321 | NM_001114520.1 | 0 | calcium/calmodulin-dependent protein kinase II inhibitor 1 |
| isotig02917 | NM_001105494.1 | 0 | cortexin 1 |
| isotig02909 | NM_001034301.1 | 0 | small proline-rich protein 3 |
| isotig02758 | XM_002704108.2 | 3.00E-82 | uncharacterized LOC100174927 |
| isotig01555 | XM_001250683.3 | 0 | carbonic anhydrase 1-like |
| isotig01197 | NM_001046002.1 | 0 | CD48 molecule |
| isotig01135 | XM_001250514.3 | 0 | family with sequence similarity 127 member |
| isotig00546 | XM_002703711.1 | 0 | uncharacterized LOC100296060 |
| HQDJLS201EQ9I0 | NM_001195051.1 | 0 | myeloma overexpressed 2 |
| HQDJLS201E0QJG | NR_031346.1 | 8.00E-37 | microRNA mir-22 |
| HQDJLS201DR0RE | NM_001101062.1 | 0 | platelet factor 4 |
| HQDJLS201DIS7E | NR_031036.1 | 6.00E-28 | microRNA mir-2299 |
| HQDJLS201D0ZF0 | XM_002688453.2 | 1.00E-136 | chromosome 6 open reading frame, human C4orf48 |
| HQDJLS201CC0TS | NM_001040587.1 | 0 | peptide YY |
| HQDJLS201C8GIX | XM_003587966.1 | 1.00E-142 | uncharacterized LOC100848866 |
| HQDJLS201BYCQT | XM_002694067.1 | 0 | uncharacterized LOC100337507 |
| HQDJLS201BB3F3 | XM_002706588.1 | 1.00E-171 | uncharacterized LOC100297713 |
| HQDJLS201B6EK0 | NR_031258.1 | 2.00E-21 | microRNA mir-2443 |
| HQDJLS201AVW6X | NR_031101.1 | 7.00E-31 | microRNA mir-2315 |
| HQDJLS201AVU7T | NR_030925.1 | 5.00E-28 | microRNA mir-484 |
| HQDJLS201AVDPM | XM_003582338.1 | 1.00E-167 | c-X-C motif chemokine 15-like |
| HNTXQRY02JB3OS | XM_003582854.1 | 1.00E-129 | uncharacterized LOC100852187 |
| HNTXQRY02IVSET | XM_002702236.1 | 1.00E-99 | uncharacterized LOC100196898 |
| HNTXQRY02ISTGH | NR_030845.1 | 4.00E-26 | microRNA mir-1940 |
| HN40NPI01E3OZ6 | NM_001037627.1 | 0 | FXYD domain containing ion transport regulator 7 |
| HN40NPI01DK4LJ | NM_175829.2 | 0 | NADH dehydrogenase (ubiquinone) 1 alpha subcomplex, 5 |
| HN40NPI01B3BQL | XM_003583208.1 | 1.00E-134 | uncharacterized LOC100851008 |
